# Universal droplet propulsion by dynamic surface-charge wetting

**DOI:** 10.1038/s41378-024-00745-x

**Published:** 2024-09-26

**Authors:** Yifan Zhou, Jiayao Wu, Ge Gao, Yubin Zeng, Sheng Liu, Huai Zheng

**Affiliations:** 1 School of Power and Mechanical Engineering, Wuhan University, Wuhan, 430072 China; 2 The Institute of Technological Sciences, Wuhan University, Wuhan, 430072 China

**Keywords:** Engineering, Materials science

## Abstract

Controllable droplet propulsion on solid surfaces plays a crucial role in various technologies. Many actuating methods have been developed; however, there are still some limitations in terms of the introduction of additives, the versatilities of solid surfaces, and the speed of transportation. Herein, we have demonstrated a universal droplet propulsion method based on dynamic surface-charge wetting by depositing oscillating and opposite surface charges on dielectric films with unmodified surfaces. Dynamic surface-charge wetting propels droplets by continuously inducing smaller front contact angles than rear contact angles. This innovative imbalance is built by alternately storing and spreading opposite charges on dielectric films, which results in remarkable electrostatic forces under large gradients and electric fields. The method exhibits excellent droplet manipulation performance characteristics, including high speed (~130 mm/s), high adaptability of droplet volume (1 μL–1 mL), strong handling ability on non-slippery surfaces with large contact angle hysteresis (CAH) (maximum angle of 35°), significant programmability and reconfigurability, and low mass loss. The great application potential of this method has been effectively demonstrated in programmable microreactions, defogging without gravity assistance, and surface cleaning of photovoltaic panels using condensed droplets.

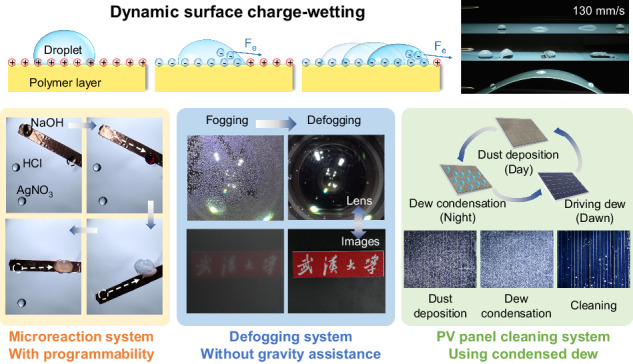

## Introduction

Droplet transport on solid surfaces is ubiquitous in nature and is related to many application technologies, such as microfluidics operation^1,2^, water harvesting^3,4^, surface self-cleaning^5^, condensation heat transfer enhancement^6,7^, energy harvesting^8,9^, and 3D printing^10^. High-efficiency droplet propulsion is necessary for facilitating the innovation and revolution of droplet-involved technologies^11,12^. Based on the requirements of various applications, the desired droplet transport should have specific performance parameters, such as high transport velocity, real-time and programmable handling, high accuracy and flexibility, unlimited distance, low droplet residue, usability for a wide range of droplet volumes, liquid materials, and solid surfaces.

Droplet motion on solid surfaces is hindered by contact angle hysteresis^13^, and droplet motion occurs only when the driving force overcomes the lateral adhesion force^14^. Fundamentally, direct droplet manipulation strategies can result from modulating the surface wettability to adjust the lateral adhesion force, which consists of building surface wettability gradients^15^ and introducing a slippery liquid-infused porous surface (SLIPS)^16^. Surface wettability gradients can be generated via surface chemical composition gradients^17,18^ and topographic micropatterns^19^. Moreover, surface wettability gradients have been widely explored and adopted due to droplet self-motion in certain ways. In addition, tuning the surface chemical composition gradients^20^ or surface topographies^21^ can realize dynamic droplet manipulation. However, these strategies have some constraints, including a limited handling distance and a lack of handling flexibility. SLIPSs can inhibit contact angle hysteresis and minimize the lateral adhesion force. With gravity, droplets with a wide range of surface tension exhibit directional and effective transport. However, the weak durability and large viscous resistance force of a lubricant-impregnated surface are the main challenges hindering its application in harsh environments, such as in those that experience vapor condensation^22^.

To increase the controllability and effectiveness of droplets, active droplet actuating strategies involving external stimuli, including thermal^23^, vapor^24^, optical^25^, acoustic and vibration^26–28^, magnetic^29,30^, and electric^31–34^ stimuli, have been developed. Thermal, vapor, and optical stimuli drive droplets by inducing an imbalance of surface tension on their surfaces or asymmetric contact angles on the surfaces of solids. Despite their convenience in implementation, these droplets have low velocities and strict material requirements for liquids and solid surfaces. Acoustic, magnetic, and electric stimuli apply forces on the droplets and result in high driving controllability. Among them, the electric field-based methods have specific advantages, including the ability to use various droplet materials^35^, not requiring additives^36^, and real-time control^37^; thus, they have high application prospects^38,39^. Electric field stimuli are applied mainly through electrowetting^40^ and dielectrowetting^41^, which are realized by constructing local and dynamic electric fields with complex electrode patterns activated by control circuits via stepwise control. They show remarkable driving forces; however, the complex control and electrode fabrication requirements weaken their serviceability. To eliminate electrodes, charged surfaces with unipolar charge gradients have recently been proposed to conveniently handle droplets at long distances^42–44^. They are promising in high-precision manipulation^45^; however, droplet manipulation has been demonstrated only on superhydrophobic or slippery surfaces with relatively small driving forces. A question has been raised that must be comprehensively considered: can electrowetting be conducted by simultaneously charging surfaces with opposite charges to boost the driving forces?

Herein, we demonstrate a universal droplet propulsion method through dynamic surface-charge wetting induced by the deposition of oscillating and opposite surface charges. On dielectric solid surfaces, strong and dynamic electric field gradients are generated through the alternate deposition of negative and positive charges in a contact-free manner. This process is initiated by corona discharge. In dynamic surface-charge wetting, droplets are being continuously and effectively transported along or against electric field lines. Due to the presence of superstrong electric field gradients along solid surfaces, the driving forces are six times greater than the droplet gravitational forces. Thus, this method can be applied to surfaces with various surface wettability, from hydrophilic to hydrophobic. In addition, droplet materials are not constrained, and both conductive and dielectric droplets are highly feasible. Moreover, the manipulation method has high flexibility in terms of tuning the modes of droplet handling (i.e., driving, trapping, and tracking) on solid surfaces. This method demonstrates high configurability and performance. Finally, powerful applications, including programmable microreactions, surface cleaning using condensed droplets, and antifogging without gravity assistance, have been successfully demonstrated.

## Results

### Droplet propulsion on diverse solid surfaces

**Fig. 1:**
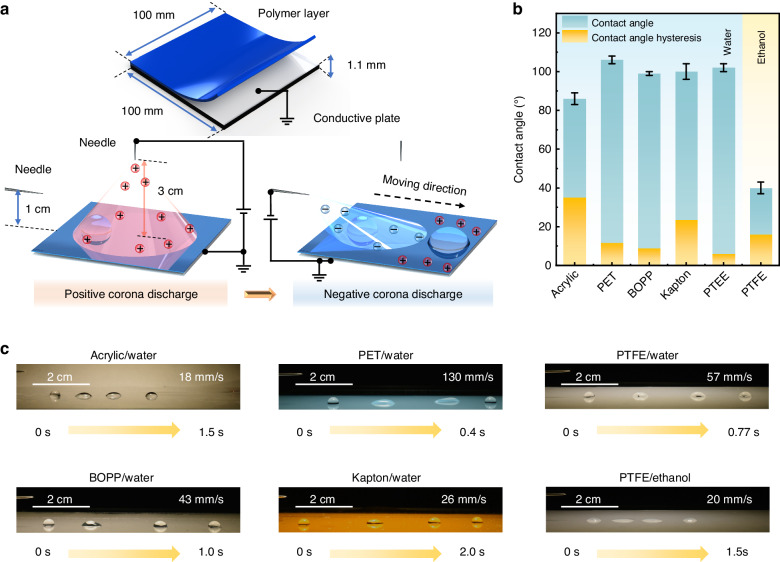
Universal droplet propulsion. **a** Schematic illustration of a droplet propulsion system consisting of polymer films, ITO-coated glasses, and needle electrodes. A polymer with a thickness ranging from 0.05 to 2 mm is pasted on the conductive surface of ITO. The vertical distances between the top needle electrode and the side needle electrode to the polymer surface are 3 and 1 cm, respectively. The horizontal distance between the two needle electrodes varies from 3 to 7 cm. The applied voltages on the top needle electrodes and side needle electrodes vary from 0 to 15 kV and from 0 to −9 kV, respectively. **b** Contact angles and CAH: DI water droplet on acrylic, PET, BOPP, Kapton, PTFE surfaces, and ethanol droplet on PTFE surface. **c** Sequential images of 10-μL droplet motions under six conditions. The sequence of applied voltages is as follows: 11 kV (10 s duration) on the top needle electrode and −7 kV on the side needle electrode

Droplet propulsion is carried out by the two-step deposition of oscillating and opposite surface charges, realized by a set of very simple equipment. Figure 1a shows an experimental scheme of the droplet propulsion system involving the deposition of opposite surface charges, which consists of indium tin oxide (ITO) conductive glass covered by a flat polymer layer and two steel needle electrodes for electrical stimulation. Portable batteries with step-up transformers are applied to safely induce corona discharge with a low current (4 μA) and low power consumption (0.1 W) around the needle tips to charge the surfaces (Figs. S1, S2; Supplementary Information). The working distance between electrodes determines the surface charge density and coverage area. This distance is optimized by performing experiments. Droplet propulsion results from two sequential charge deposition processes. First, the vertical needle electrode in the middle charges the surface for 10 s^46^. Subsequently, the horizontal needle electrode parallel to the polymer layer charges the surface with opposite charges. Afterward, the droplet is immediately transported far from the electrode. The effective distance for lateral electrode-driven droplets reaches ~2 cm. Notably, if only one charge deposition step is carried out, the droplet cannot be propelled (Fig. S3; Supplementary Information).

This droplet propulsion technique shows high universality in various surfaces and liquids, even on non-slippery surfaces. Five kinds of common polymer layers—acrylic, polyethylene terephthalate (PET), biaxially oriented polypropylene (BOPP), Kapton, and polytetrafluoroethylene (PTFE)—and two kinds of liquid droplets—water and ethanol—are examined for droplet propulsion. As shown in Fig. 1b, their contact angles vary from 40° to 106°, and the maximum contact angle hysteresis reaches 35° (Fig. S4; Table S1; Supplementary Information). As shown in Fig. 1c, both the water and ethanol droplets can be effectively propelled on different surfaces. The droplet starts to move and quickly gains a large velocity; after moving several centimeters, the velocity quickly decreases to zero (Fig. S5; Supplementary Information). The fluorescence images indicate that a low amount of droplet residue is left on the surface (Fig. S6; Supplementary Information), and the lossless propulsion property is important for chemical/bioassays. The minimum average velocity occurs on the acrylic surface (18 mm/s), and the water droplet on the PET surface has the largest average velocity (130 mm/s). The velocity is related to the contact angle hysteresis (Fig. S7; Supplementary Information), and a smaller contact angle hysteresis leads to a higher velocity. Moreover, nonconductive silicone oil droplets can be propelled (Fig. S8; Supplementary Information). In addition to the above conditions, droplet propulsion can be realized on the surfaces of everyday objects, such as plastic gloves, glasses, wrapping papers, and woodwares (Fig. S9; Movie S1; Supplementary Information). The mechanism shows that the conductive layer material is highly adaptable. Various conductive layers are tested: ITO glass, graphite plate, copper foil, and tin foil layers. Conductive materials with different mechanical properties enable multifunctional droplet performance. Soft copper foil and tin foil enable curved surfaces and flexible electrode applicability. In addition, droplet propulsion shows high repeatability, and more than 100 stable and consistent experiments using each type of dielectric film prove the reliability and reproducibility of the operating principle.

### Dynamic surface-charge wetting and its mechanism

**Fig. 2:**
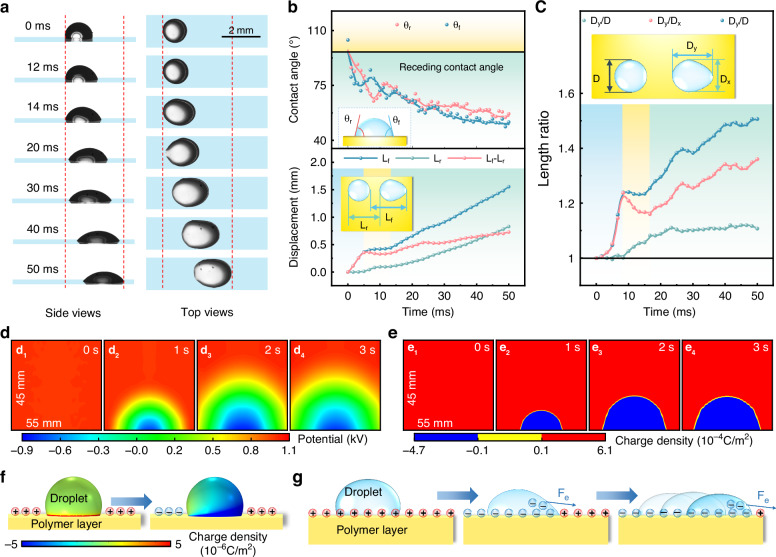
Mechanism of dynamic surface-charge wetting. **a** Side-view and top-view sequential images of the dynamics of a 1-μL water droplet within 50 ms. The sequence of applied voltages is 11 kV (10 s duration) on the top needle and −7 kV on the side needle. **b** Time-dependent rear and front contact angles of the droplet (*θ*_r_ and *θ*_f_, respectively), the simultaneous displacement of the rear endpoint and front endpoint of the droplet (*L*_r_ and *L*_f_, respectively), and the difference in *L*_f_ − *L*_r_. **c** Dimensional ratios of the droplet in two directions (*D*, *D*_*y*_, and *D*_*x*_ are the initial diameter, the length in the droplet motion direction and the length in the perpendicular direction, respectively). **d** Electric potential map evolution on the PET surface at different durations (d_1_-d_4_: 0, 1, 2, and 3 s) of negative corona discharge after positive corona discharge. **e** Surface-charge density evolution on the PET surface at different durations (e_1_-e_4_: 0, 1, 2, and 3 s) of negative corona discharge after positive corona discharge. **f** Droplet charge distribution when it is located at the only positively charged surface and the boundary of opposite charge polarity. **g** Schematic illustration of the mechanism of droplet propulsion. This schematic includes the changes in charges and droplet dynamics caused by the electrostatic force *F*_e_

Compared to electrowetting propulsion^47^, droplets undergo continuous transport with front contact angles that are smaller than rear contact angles. The side-view sequential images in Fig. 2a show that the right side of the three-phase contact line spreads toward the right side, while the left side remains pinned (Movie S2; Supplementary Information). The rightward spreading of the contact line leads to a continuous and simultaneous decrease in the front and rear contact angles. When each contact angle decreases to ~72° (less than the receding contact angle), the left contact line starts to move to the right. Afterward, the droplet is continually transported as the contact angle gradually decreases (Fig. 2b). In contrast to isotropic spreading during electrowetting, the droplet presents anisotropic dynamics (see the top-view images in Fig. 2a). The droplet contact area increases omnidirectionally, and the maximum diameter is ~1.5 times greater than the initial diameter (Fig. 2c). In contrast, the diameter in the *Y* direction is significantly greater than that in the *X* direction. The ratio, *D*_*y*_/*D*_*x*_, is 1.3.

Droplet propulsion results from dynamic charge transport and surface potential changes on surfaces during the deposition of oscillating and opposite surface charges. The surface-charge pattern evolution can be detected by an off-line electrostatic voltmeter^46^ (Fig. S10; Supplementary Information). After the surface is charged by the top positive needle electrode, it is found that the detected area (45 × 55 mm) has a uniform and high surface potential (~1.1 kV) (Fig. 2d_1_). After subsequent negative charge deposition from the left side, a negative potential occurs on the previously positively charged surface. Figure 2d_2_–d_4_ shows the evolution of the surface potential patterns with the charging time. The negative charge on the surface exhibits a semielliptical pattern with a minimum value of approximately −0.9 kV, and the pattern propagates on the surface as the deposition duration increases until it finally stabilizes (Fig. 2d_3_, d_4_). According to the measured surface potentials, the surface-charge density *σ* can be calculated by the following equation^48^:

1 $$\sigma ={Uc}$$

2 $$c=\varepsilon {\varepsilon }_{0}/d$$

where *U* is the surface potential, *c* is the capacitance per area of the PET layer, and $\varepsilon {\varepsilon }_{0}$ and $d$ are the permittivity and thickness of the PET layer, respectively. Using the finite element method, we can obtain the charge density distributions (Fig. 2e). The initial surface charge is positive, and the density reaches 6.1 × 10^−4^ C/m^2^. Under negative corona discharge conditions, negative charges are deposited on the PET surface, and the negative charge pattern spreads with increasing deposition time. The charge distributions indicate that the deposited charges on the surface can be neutralized, and charges with opposite polarities can be redeposited. These processes enable reconfigurable droplet manipulation because the old surface charge density gradient can be freely removed and replaced by a new gradient on a single film. To analyze the electrostatic force acting on the droplet, we simulate the charge distribution of the droplet surface during the charging process (Figs. S11, S12; Supplementary Information). Initially, the droplet only carries positive charges, which are mostly distributed at the bottom area, and it becomes negatively charged when the negative charge pattern spreads to the droplet position (Fig. 2f). Noticeably, the charge is concentrated on the front edge and the bottom surface of the droplet. Therefore, there is a large electric field at the front edge of the three-phase contact line, which results in a large electrostatic force on the droplet at the boundary of the opposite charge polarity to overcome the large surface pinning force; thus, dynamic surface-charge wetting is achieved, as schematically shown in Fig. 2g.

**Fig. 3:**
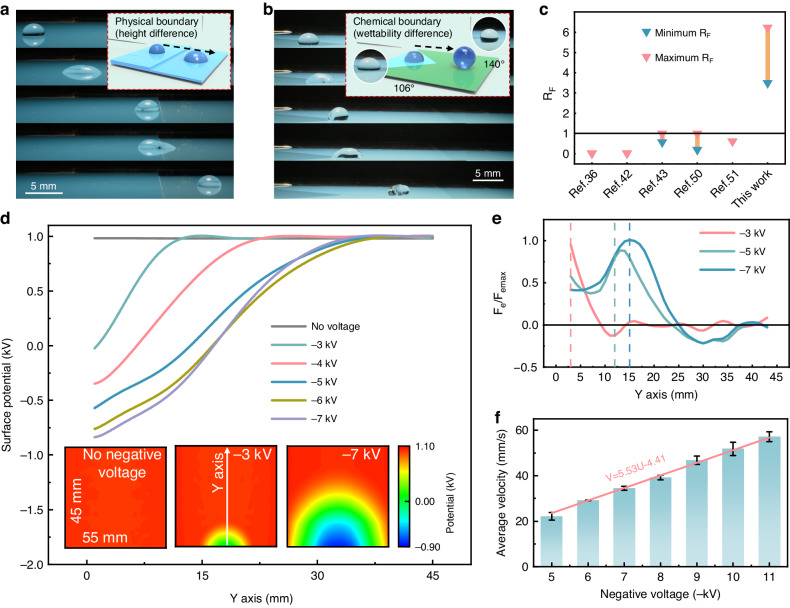
Remarkable electrostatic force for droplet propulsion. **a** Sequential images of a 15-μL water droplet overcoming a step-edge structure. The inset is a schematic illustration of droplet motion. The sequence of applied voltages is as follows: 11 kV (10 s duration) on the top needle and −9 kV on the side needle. **b** Sequential images of a 20-μL water droplet anti-wettability gradient. The inset is a schematic illustration of droplet motion considering the equilibrium contact angles of two regions. The sequence of applied voltages is as follows: 11 kV (10 s duration) on the top needle electrode and −9 kV on the side needle electrode. **c** Comparison of the ratio of droplet driving force to droplet gravitational force (*R*_F_) between this work and the reported approaches. **d** Surface potential at the *Y*-axis at different negative voltages applied on the side needle electrode. The inset is the surface potential mapping variation at different negative voltages applied on the side needle electrode. **e** Ratio of the electrostatic force *F*_e_ to the maximum electrostatic force *F*_emax_ at different *Y* positions when different negative voltages are applied on the side electrode. **f** Average velocity variations at different negative voltages on the side needle electrode. A voltage of 11 kV (10 s duration) is applied on the top needle

Dynamic surface-charge wetting can generate remarkable driving forces to overcome the challenges associated with droplet transport. Research has been conducted on intercepting droplets by using the edge effect or allowing droplets to spontaneously move along the wettability gradient; however, it is usually challenging to overcome the effects of the edge or wettability gradients. Figure 3a, b displays the process by which droplets move through an anti-edge and anti-wettability gradient by the deposition of oscillating and opposite surface charges (Movie S3; Supplementary Information). The micropipette-based method^49^ is used to measure the resistance force at the barrier, which is ~3 times greater than its gravitational force. Considering the lateral adhesion force and friction force, we obtain the maximum electrostatic force *F*_emax_ (Figs. S15, S16; Table S2; Supplementary Information). We evaluate the force magnitude compared with recent electric stimulation methods. Considering that the droplet volume can affect the force, the ratio of the driving force to the droplet gravitational force *R*_F_ is introduced; our method displays a much larger ratio than other methods^36^^,^^42^^,^^43,50,51^ (Fig. 3c).

The electrostatic force distribution can be regulated by adjusting the applied voltage on the side needle. As shown in Fig. 3d, the surface potential clearly varies with negative voltage. The negative potential areas increase as the applied negative voltage increases (all the measurements are based on the saturated charging state), and the opposite charge boundary moves forward simultaneously. Furthermore, the electrostatic force has a different distribution, and the maximum force position moves forward as the voltage increases (Fig. 3e). The charge polarities of the droplet and its nearby area influence the force distribution, the opposite charge boundary location, instead of the highest potential location, is the *F*_emax_ position. The ratio of *F*_e_ to *F*_emax_ can be negative in some places (Fig. S14; Supplementary Information). Therefore, under a larger applied voltage, a larger electrostatic force acts on droplets for a longer distance, which results in an apparent relationship in which a larger voltage leads to a higher average droplet velocity (Fig. 3f).

### High-performance droplet handling

**Fig. 4:**
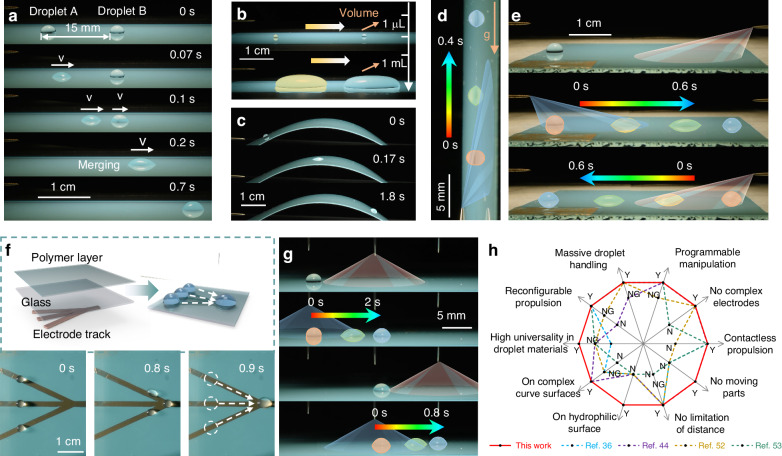
High-performance droplet handling. The needle electrode arrangements in (**a**–**d**, **f**) are the same as that in Fig. 1a. The sequence of applied voltages is as follows: 11 kV (10 s duration) on the top needle electrode and −7 kV on the side needle electrode. The red and blue cone patterns in (**d**–**f**) denote positive corona discharge and negative corona discharge, respectively. **a** Sequential images of two 10-μL droplets moving and merging. **b** Time-lapse trajectory of a 1-μL and a 1-mL water droplet moving forward. **c** Side-view sequential images of a 3-μL droplet moving on a curved PET surface. **d** Side-view sequential images of a 2-μL droplet moving on a vertical PET surface. **e** Sequential images of a 15-μL droplet moving from one side to the other and moving back under two opposite needle electrodes. The sequence of applied voltage is as follows: 9 kV (10 s duration) on the right needle electrode and −9 kV (2 s duration) on the left needle electrode. **f** Top-view sequential images of three 7-μL droplets moving along the preset track and merging. The inset is a schematic illustration of the assembly. **g** Sequential images of the sustainable motion of 7-μL droplets under digitally applied voltages of three vertical needle electrodes. A voltage of 4 kV (5 s duration) is applied to the needle, and then the droplet moves to its bottom after a voltage of −4 kV (5 s duration) is applied to the needle behind it. The droplet can continuously move forward when this process is repeated. **h** Comparison between the dynamic surface-charge wetting and previously reported electric-based droplet manipulation from ten items. “Y”, “N”, and “NG” denote yes, no, and not given in the literature, respectively

Remarkable driving forces and contactless stimuli in surface-charge wetting allow versatile droplet manipulation. The synchronism of droplet propulsion and charge spreading on surfaces endows a sequential droplet driving function that benefits droplet merging (Fig. 4a). The large driving force greatly expands the applicable droplet volume range. Droplets with volumes ranging from 1 μL to 1 mL can be propelled long distances (Fig. 4b). In addition, contactless charge deposition and remarkable driving forces facilitate droplet transport with excellent spatial structure adaptability. Droplets show continuous motion on curved and vertical surfaces against the force of gravity (Fig. 4c, d).

Surface-charge wetting involves highly programmable and reconfigurable droplet manipulation through spatially and temporally controlling the charge deposition. Temporal charge deposition control enables high efficiency and lossless propulsion. By alternately applying positive and negative voltages on two-directional opposite needle electrodes (left and right), the droplets can move back and forth, and there is no residue (Fig. 4e). The spatial charge deposition enables on-demand droplet motion. We design a trident electrode pattern beneath the polymer layer, and the charge only accumulates where the electrode exists; thus, three droplets move along their track and coalescence (Fig. 4f). In addition, spatially and temporally combined charge deposition control promotes droplet transport over an unlimited distance. We use multi-vertical needle electrodes to generate the oscillating deposition of opposite surface charges in a relayed manner, propelling the droplets to move under the next needle electrode (Fig. 4g). By scaling needle electrodes, the droplet can move over a long distance. In addition, the spatiotemporal charge deposition enables the droplet to change its moving direction at will. We use three orthogonal needle electrodes to sequentially introduce charge deposition that oscillates in different directions so that the droplet moves and swerves (Fig. S21). The mechanism of the above process is schematically shown in Figs. S17–S21 in the Supplementary Information. The above multifunctional droplet propulsion can be achieved on a single film due to reconfigurability. Combined with high-performance droplet handling (Movie S4; Supplementary Information), dynamic surface-charge wetting performs much better in various aspects of droplet handling than other reported droplet propulsion methods^36,44^^,^^52^^,53^ (Fig. 4h).

### Versatile droplet propulsion applications

**Fig. 5:**
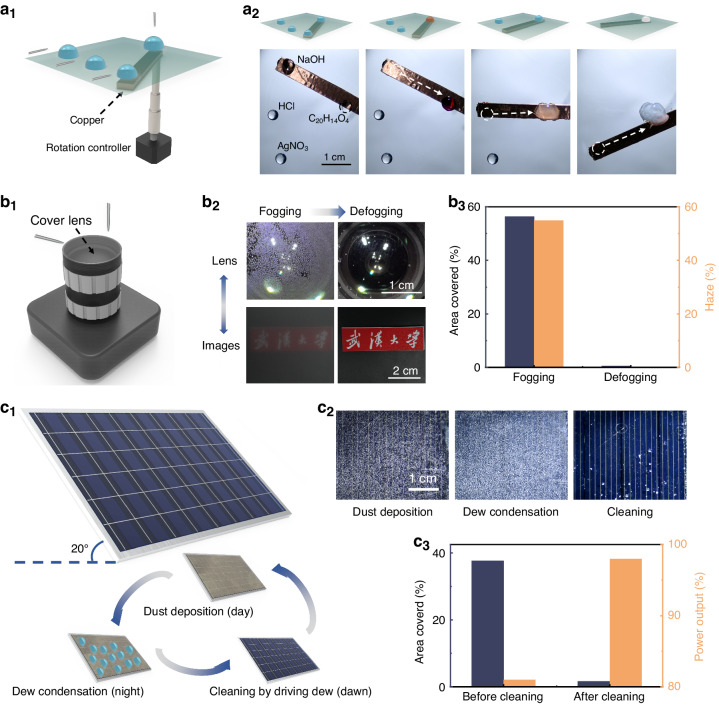
Versatile droplet propulsion applications. **a** Schematic illustration of the programmable and sequential microreaction assembly (a_1_). Sequential images of programmable and sequential microreactions (a_2_). The applied voltages on the top needle and side needle are 11 and −7 kV, respectively. **b** Schematic illustration of the defogging assembly (b_1_). Sequential images of defogging and corresponding images taken by the camera (b_2_). The fog coverage areas and haze before and after defogging (b_3_). The applied voltages on the top needle electrode and side needle electrode are 11 and −9 kV, respectively. **c** Schematic illustration of PV panel surface cleaning by driving dews (c_1_). Sequential images of PV panel surface cleaning by driving dews (c_2_). The dust coverage area and the power output before and after defogging (c_3_). The applied voltages on the top and side needle electrodes are 11 and −9 kV, respectively

The sequential control ability, adaptability of small droplets, and resource reduction ability of surface charge have been proven through programmable droplet microreactions, PV surface cleaning using dews, and antifogging on lenses (Movie S5; Supplementary Information). In microchemical detection, lossless and sequential control of multistep droplet coalescence is necessary; however, previous strategies require repeated movement of the tweezer component, which is tedious and inefficient. We aim to achieve high efficiency programmable and sequential droplet microreactions simply with a rotatable conductive bar. Because the charges can only be deposited on the area where the conductive bar is located under the film, by depositing oscillating and opposite surface charges, only connected droplets can merge, while other droplets remain static. By introducing a controller to modulate the bar position to connect different droplets, charges are deposited in sequence on different areas, droplets sequentially move onto the conductive bar, and multistep droplet coalescence is achieved (Fig. 5a). The detailed and schematic steps are shown in Fig. S22 in the Supplementary Information. Due to the ability to propel microdroplets, this manipulation method can be used for eliminating fog. Herein, we demonstrate the defogging function of the camera lens. Previous electrical stimulus methods involve arranging the electrode pair below the lens, which inevitably increases the risk of electrical breakdown^54,55^. By depositing oscillating and opposite surface charges, we simply place a grounded conductive plate under the lens with a relatively low electrical breakdown risk and no gravity assistance. This method shows excellent defogging performance; the fog coverage ratio decreases from 56.3% to 0.5%, and the haze decreases from 54.9% to 0.2% (Fig. 5b). In the field of solar power generation, dust accumulation on PV panels is an issue that decreases the energy conversion efficiency^56^. We realize dust cleaning by manipulating the condensed droplets generated at night (Fig. 5c_1_). We simulate the condition in which dews are formed on PV panels with accumulated SiO_2_ particles. With the oscillating deposition of opposite surface charges, the dews quickly merge and slide off, the dust coverage ratio decreases from 37.6% to 1.6%, and the power output increases by 17% (Fig. 5c_3_).

## Discussion

In summary, a unique and universal droplet propulsion method by dynamic surface-charge wetting is presented. This technique involves the deposition of oscillating and opposite surface charges. This method has the advantages of a large driving force (~6 times larger than the force of gravity) and easy implementation ability; thus, it can overcome the inherent limitations of conventional methods, which can require surface treatments, droplet additives, and moving part dependencies. The deposition of oscillating and opposite surface charges creates variable surface charge patterns, highly flexible transport abilities, and large driving forces. This process increases the versatility of droplet manipulation, allowing for high-speed propulsion (~130 mm/s), droplet merging, large-volume droplet manipulation (1 μL–1 mL), droplet climbing, infinite distance transport, low-residue and repeatable manipulation, and predetermined trajectory movement. Based on these powerful functions, the application potential of the propulsion method has been demonstrated in programmable microreactions, antifogging without gravity assistance, and self-cleaning using condensate droplets. The high efficiency, simple implementation ability, and universal suitability of droplet propulsion can allow for the practical application of this method in a variety of fields, in biochemical research, in the surface cleaning of solar panels, and in water harvesting.

## Methods

### Material

The following materials were used: ITO-coated glass (100 mm × 100 mm × 1.1 mm, Xiangcheng Technology, STN, China); acrylic film (100 mm × 0.2 mm); PET film (50 mm × 0.06 mm); BOPP film (45 mm × 0.05 mm, JL1760); Kapton film (50 × 0.1 mm, PI100); PTFE film (100 mm × 0.1 mm); DI water (CD433277, Codow, China); ethanol (75%); silicone oil (PMX-200, 50cs, Dow Corning, USA); NaOH (0.1 mol/L); C_20_H_14_O_4_ (1 g/L); HCl (1 mol/L, HUSHI); AgNO_3_ (0.05 mol/L); SiO_2_ particles; and superhydrophobic coating material (sf-04172, SOFT99).

### Droplet manipulation

Rechargeable lithium-ion batteries (Chenke, 12 V-18650, China) and high-voltage power modules (Corso Electronic, KDHM-Q-12S15000P-VI, China) were applied to the steel needle electrodes to introduce corona discharge. Droplets were placed on various films. Then, positive and negative voltages were successively applied on the top and side needle electrodes. Fluorescence microscopy (Nikon, ECLIPSE Ni) was used to measure the droplet residue.

### Contact angle measurement

The equilibrium, advancing, and receding contact angles were measured by a contact angle system (KRÜSS, DSA25B). The measurements were all carried out with the newly prepared films pasted on ITO-coated glass without charge deposition. The equilibrium contact angles were measured using 5 μL droplets on a horizontal plate. The advancing and receding contact angles were measured using the tilting plate method with 50 μL water droplets and 20 μL ethanol droplets. With the tilting angles slowly increasing from 0°, the lower and upper contact angles when the droplet began sliding were considered the advancing and receding contact angles, respectively. The dynamic contact angle of each droplet during its initial motion was measured using ImageJ software.

### Droplet dynamics measurement

Images of droplets were obtained using a Nikon D7200 camera to observe their dynamics. Images that showed the initial droplet dynamics were obtained using a high-speed camera (MIRO C210, Phantom, USA). The displacement and velocity were obtained using Tracker software based on the videos captured by a camera. Unless stated otherwise, the displacement was obtained by analyzing the right endpoint of the droplet.

### Electric parameter measurements

The surface potentials of charged films were measured off-line using an electrostatic voltmeter (341B, Trek, USA). After the samples were subjected to positive or negative loading, charges were sequentially deposited on the films. The surface potential distribution within an area of 45 × 45 mm was obtained after the probe was scanned. The potential of the left part of the needle electrode was copied to obtain the potential of the right part because of the symmetric experimental assembly. Then, the potential distribution within an area of 45 × 55 mm was obtained. The corona discharge current was measured using a high-precision ammeter (Keithley, 6517B, USA). The input of the ammeter was connected to the ITO conductive surface, and the output of the ammeter was grounded. When a voltage was applied to the needle, there was a relatively stable current. The output power of the PV panel was obtained by measuring the current and voltage using an ammeter (Keithley, 6517B, USA). The power was calculated by the following equation:

3 $$P={UI}$$

where *P* is the output power of the PV panel and *U* and *I* are the measured output voltage and current, respectively.

### Coverage ratios of particles and droplets

A Peltier cooling system is used to form fog and dew. The coverage ratios of the particles and droplets were measured using ImageJ software.

## Supplementary information

Revised Supplementary Information-Clean Version

Supplementary Movie S1

Supplementary Movie S2

Supplementary Movie S3

Supplementary Movie S4

Supplementary Movie S5

## Data Availability

All data are available in the main text or in the Supplementary Information.
