# Supplementary material for: Universal droplet propulsion by dynamic surface-charge wetting
Source: Microsyst Nanoeng. 2024 Sep 26;10:134. doi: 10.1038/s41378-024-00745-x (PMC11427456; doi:10.1038/s41378-024-00745-x)
Supplement: Supplementary file 1 — Revised Supplementary Information-Clean Version [file 41378_2024_745_MOESM1_ESM.pdf]

Supplementary Information

Universal Droplet Propulsion by Dynamic Surface-charge Wetting

Yifan Zhou[1], Jiayao Wu[2], Ge Gao[1], Yubin Zeng[1], Sheng Liu[1,2]*, and Huai Zheng[1,2]*

[1]School of Power and Mechanical Engineering, Wuhan University, Wuhan 430072, China

[2]The Institute of Technological Sciences, Wuhan University, Wuhan 430072, China

E-mail: huai_zheng@whu.edu.cn (H. Z.); victor_liu63@126.com (S. L.)

1. Experiment details

1.1 Additional experiment description

The experiment assembly is demonstrated in Figure S1. Apart from the main part of experiment system, which contains the polymer layer, conductive plate, and needles, the other equipment, cameras and electric source are also shown. The applied voltage is supplied by a system consisted of battery and voltage amplifier, which guaranteed the portability and security of droplet steering. Though the applied voltage could reach about 15 kV, it is noted that the current of needle is only about 9 μA, which is measured and shown in Figure S2. The droplet dynamics observation and analysis are based on two orthogonal cameras which record the side view and top view droplet images, then the droplet locations at different times are gained by analyzing the movies using software Tracker.

The sequential positive and negative applied voltages on two needle electrodes are necessary, which is corresponding with our mechanism that deposition of oscillating and opposite surface charges propels droplet. With only positive and negative applied voltage, the droplet shows no respond, but when there are sequential positive and negative applied voltages, droplet could move forward (Figure S3). Figure S5 shows the time-dependent displacement and velocity of droplet on PET surface. The droplet motions could be concluded that the droplet starts to move and quickly gains a large velocity, after moving several centimeters, the velocity soon decreases to zero. The acceleration process is owing to a large electrostatic force, which overcomes the lateral adhesion force and supplies droplets with high speed. The deacceleration process results from two effects. A droplet with a large moving velocity will be subjected to a large friction force. In addition, after leaving the boundary of opposite charges, the droplet is subjected to a smaller even negative propulsion force. We also examine the droplet mass loss during transport by fluorescence imaging using a fluorescence microscope (Nikon, ECLIPSE Ni). Compared the fluorescence images before and after droplet propulsion, low fluorescence reflection is detected, which indicates that there is low droplet residue during transport (Figure S6).

Universal droplet propulsion has nothing to do with the material, electric conductivity, and viscosity of droplet. More surfaces and droplets test results are shown in the following. The contact angles are shown in Figure S4. It is noted that owing to the different micro structure and chemical component of polymer surface, there is different contact angle hysteresis (CAH), which leads to different velocity of droplet motion. The relation is shown in Figure S7, the average velocity is larger when CAH is smaller. Apart from above droplet motion, the silicone oil droplet

motion is obviously different from others (Figure S8). There is residual in the droplet motion track due to its high viscosity. It proves its ability of driving high viscosity and nonconductive droplet. Furthermore, the universal droplet motion is demonstrated intuitively by propelling droplet on common objects in daily life (Figure S9).

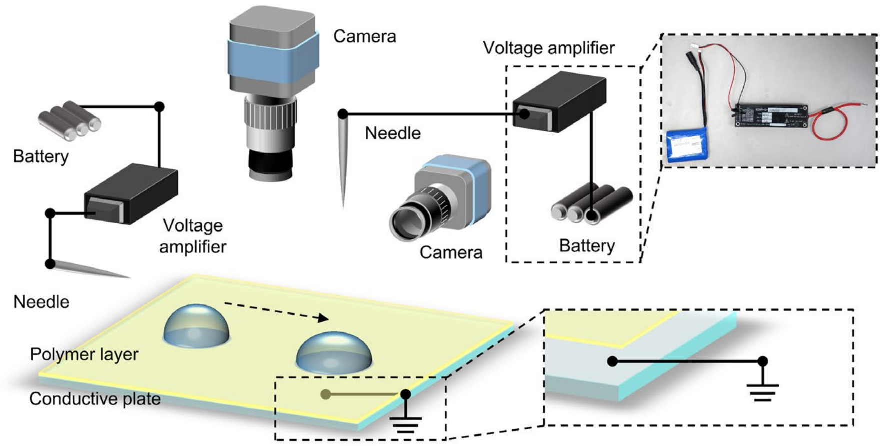

Figure S1. Schematic of whole experimental assembly.

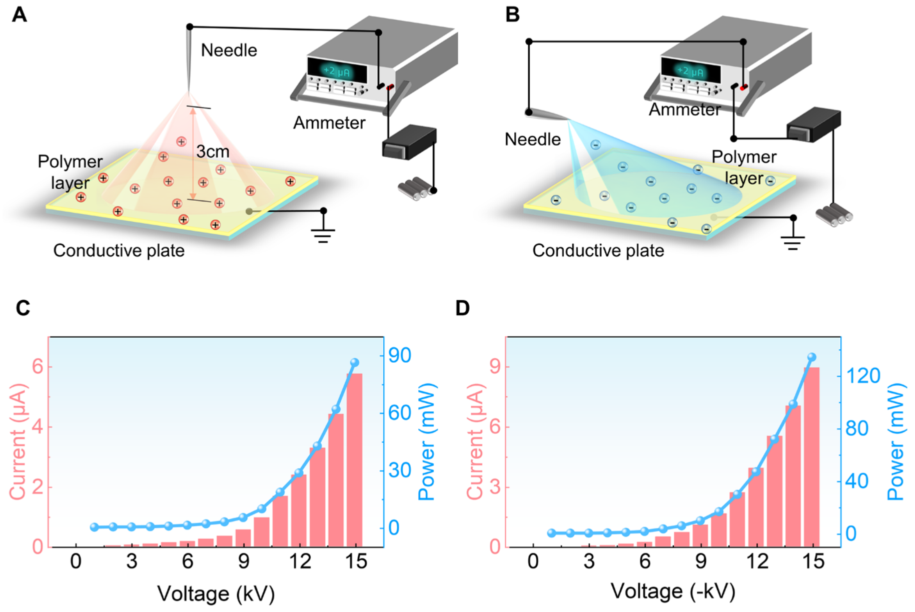

Figure S2. Current and power measurement. (A) Schematic of online testing of the current of the on the top needle electrode when positive voltage is applied. (B) Schematic of online testing of the current of the on the side needle electrode when negative voltage is applied. (C) The current and power variation at different positive

voltages applied on the top needle electrode. (D) The current and power variation at different negative voltages applied on the side needle electrode.

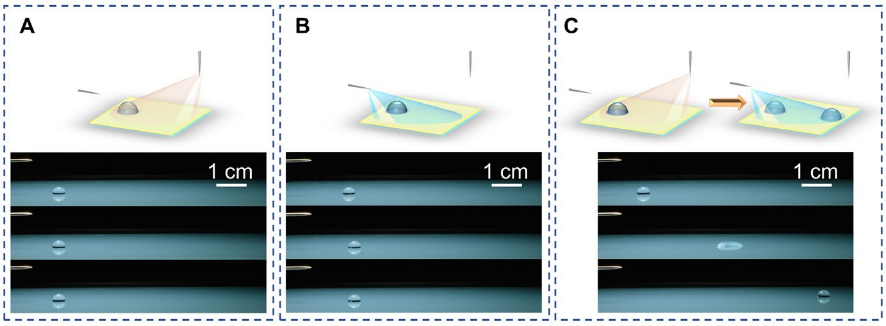

Figure S3. Necessity of two step corona discharge with opposite polarity. (A) The schematic illustration (top) and sequential images (bottom) of droplet motion when there is only applied voltage (11kV) on the top needle. (B) The schematic illustration (top) and Sequential images (bottom) of droplet motion when there is only applied voltage (-9 kV) on the side needle. (C) The schematic illustration (top) and sequential images (bottom) of droplet motion when there is sequential applied voltage (11kV) on the top needle, and applied voltage (-9 kV) on the side needle. The droplet volumes are all 10μL.

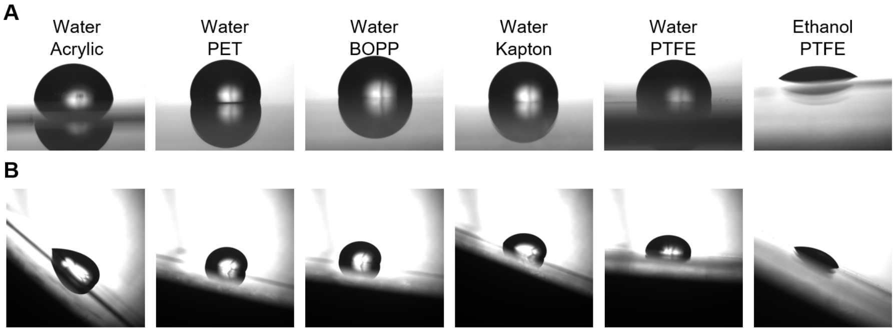

Figure S4. Contact angles and CAH measurements with angle values listed in Table S1. (A) The images of equilibrium contact angles. The droplet volumes are 5 μL. (B) The images of measuring advancing and receding contact angles using tilting plate method[1]. Water droplet on acrylic surface shows a maximum CAH of about 35°.

The ethanol droplet volume on PTFE is 20 μL, other droplet volumes are 50 μL. The values of CAH and the calculated lateral adhesion force are also listed in Table S1.

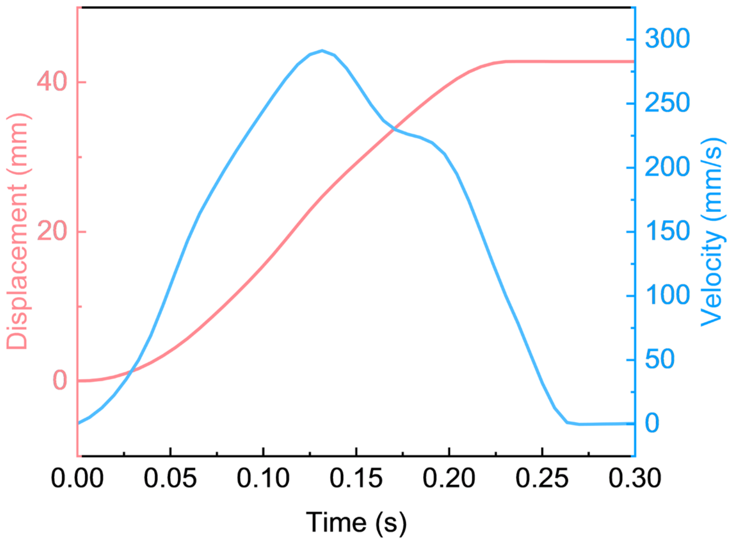

Figure S5. The time-dependent displacement and velocity of droplet on PET surface.

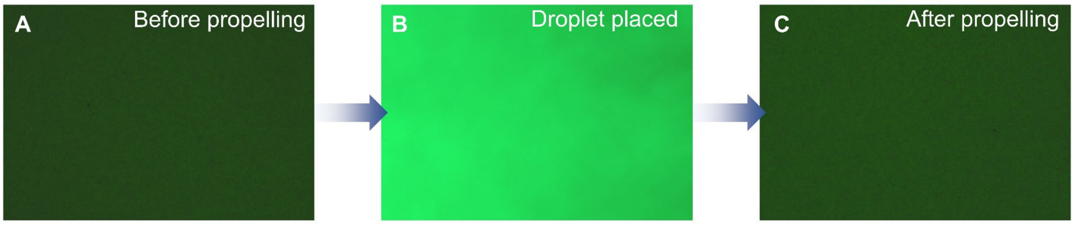

Figure S6. The measurement of droplet mass loss during transport. (A) The surface fluoroscopic image before droplet propulsion. (B) The surface fluoroscopic image with a droplet placed on the surface. (C) The surface fluoroscopic image after droplet propulsion.

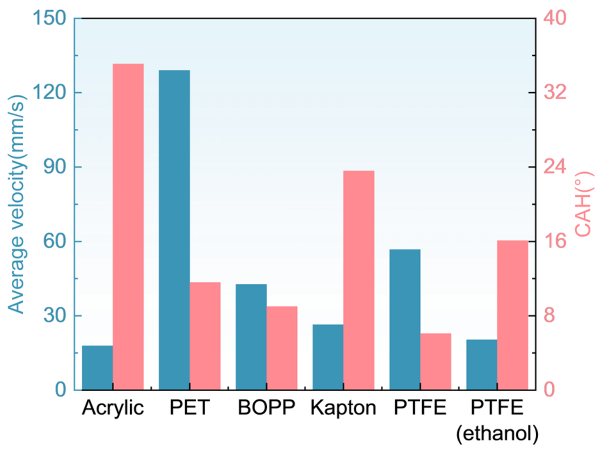

Figure S7. Relation between CAH and droplet average velocity.

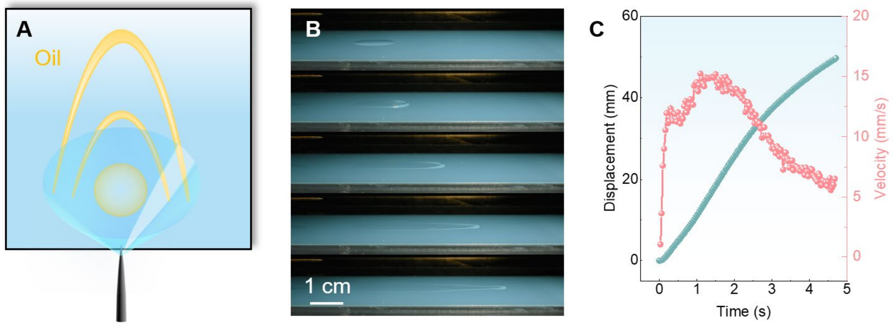

Figure S8. Propulsion of a silicone droplet. (A) Schematic illustration of silicone oil droplet motion. (B) Sequential images of a 10-μL silicone droplet motion on PET. (C) The time-dependent displacement and velocity of silicone droplet.

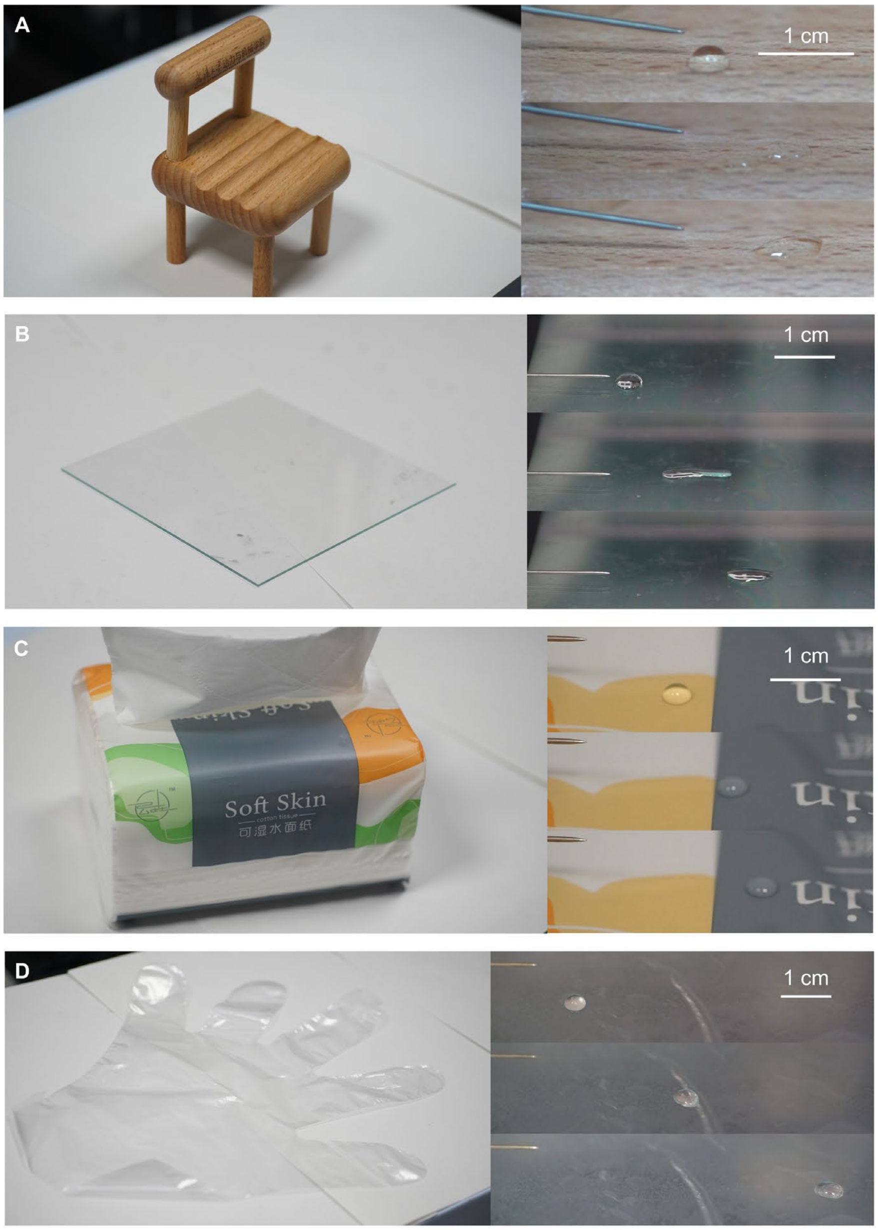

Figure S9. Droplet propulsion on everyday-object surfaces (droplet volumes are all 10 μL). (A) Woodware surface. (B) Glass surface (C) Wrapping paper surface. (D) Plastic glove surface.

1.2 Surface potential dynamics measurement

Accurate surface potential measurement intuitively demonstrates the process of polymer surface potential evolution. The potential is measured using an offline

electrostatic voltmeter[2], as shown in Figure S10. A probe with a resolution ratio of less than 1 mm connected to an electrostatic voltmeter (341B, Trek, USA) is driven by a x-y stage, then 2D surface potential distributions are obtained after the probe scans over the measured region with charges deposited.

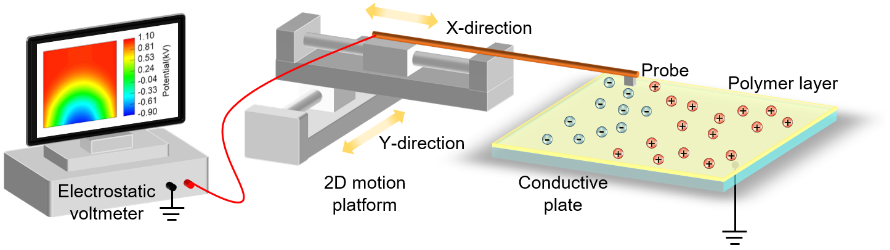

Figure S10. Schematic illustration of surface potential measurement.

2. Numerical calculation and analysis of electric force

2.1 Principle of electrostatic simulations

The electrostatic model was built using the software COMSOL to calculate the surface charge distribution and the electrostatic force. The principle of surface charge simulation is based on the following equation[3]:

$$\boldsymbol{E} = -\nabla V \tag{1}$$

$$\nabla \cdot \boldsymbol{D} = \rho_v \tag{2}$$

$$\boldsymbol{D} = \varepsilon_0 \varepsilon_r \boldsymbol{E} \tag{3}$$

where $\boldsymbol{E}$ is the electric field strength vector and V is the electric voltage. $\boldsymbol{D}$ is the electric displacement vector, ε_0 is the vacuum permittivity, ε_r is the relative permittivity, ρ_v is the bulk charge density.

Based on the above equations, the electric field and potential distributions could be calculated. According to Gauss's law, the surface charge density at a material interface equals the jump in the normal component of the displacement field.

$$\boldsymbol{n} \cdot (\boldsymbol{D_2} - \boldsymbol{D_1}) = \rho_s \tag{4}$$

where $\boldsymbol{n}$ is the normal vector at the material interface. $\boldsymbol{D_1}$ and $\boldsymbol{D_2}$ are the electric displacement vectors into and through the material interface respectively. ρ_s is the surface charge at the material interface.

The Maxwell stress tensor $\boldsymbol{T}$ is given by the following:

$$\boldsymbol{T} = \boldsymbol{E}\boldsymbol{D}^T - \frac{1}{2}(\boldsymbol{E} \cdot \boldsymbol{D})\boldsymbol{I} \tag{5}$$

where $\boldsymbol{T}$ is the Maxwell stress tensor. $\boldsymbol{E}$ is the electric field strength matrix. $\boldsymbol{D}$ is the electric displacement matrix. $\boldsymbol{I}$ is the identity matrix.

Through the integral over a closed surface surrounding the droplet, we can obtain the force $\boldsymbol{F_e}$ acting on the droplet.

$$F_e = \oint T \cdot n dA \quad (6)$$

where $\boldsymbol{F_e}$ is the electrostatic force. $\boldsymbol{T}$ is the Maxwell stress tensor. $\boldsymbol{n}$ is the normal vector at the closed surface. A is a tiny area of a closed surface.

2.2 Simulation model and boundary condition

The simulation model geometry is shown in Figure S11A. It could be divided into 3 parts, droplet, film and space. A spherical cap with a height of 1.455 mm and a radius of 1.071 mm represents a water droplet. The bottom radius of the droplet under these conditions is 1.5 mm and the contact angle is 106°. The PET film is represented by a cuboid of 55 mm × 45 mm × 0.06 mm. Droplet and film are placed in an air space of 100 mm × 100 mm × 100 mm. The boundary condition is shown in Figure S11B. The electric potential boundary conditions are using. The electric potential of upper surface is measured surface potential of PET (Figure 2D). The potential of droplet is the same as the potential at the left end point of the circle in contact with the droplet on the surface of the film. This assumption is established based on the measured potential result that the potential of left end point of the circle in contact with the droplet on the surface of the film firstly makes a difference. The lower surface of the film was set to the electrical grounding condition.

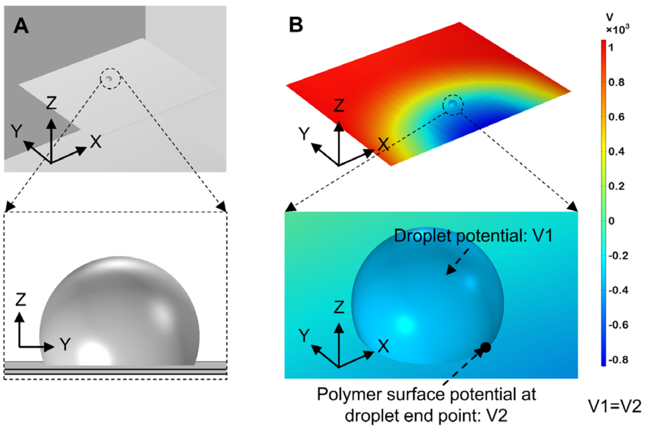

Figure S11. Simulation model and boundary condition. (A) The simulation model geometry. (B) The potential condition of polymer surface and droplet surface.

2.3 Simulation result

In order to prove our mechanism that the dynamic charge transport and surface potential evolution on surfaces during charge deposition of oscillating and opposite surface charges, the surface charge distribution of polymer surface and droplet surface is simulated on two conditions under which there is no negative voltage applied and there is negative voltage applied so that surface negative potential pattern just reached the leftend point of droplet. Therefore, the measured potential distribution of only positive applied voltage (Figure $2D_1$) and negative applied voltage with a 2-s duration after positive voltage applied (Figure $2D_2$) are using to represent the two conditions respectively. The simulated result shows that when there is only positive voltage applied, the whole polymer surface accumulated only positive charges with the density of about 5×10^{-4} C/m^2. Droplet also carries lots of positive charges which are accumulated on bottom region (Figure S12A). When there is negative voltage applied with a 2-s duration after positive voltage applied so that surface negative potential region just reaches to the left endpoint of droplet, the result shows that the surface negative charges deposited region also just reaches the droplet. Droplet carries lots of negative charges which are accumulated on the front and bottom region (Figure S12B).

The electrostatic force is then calculated. The maxwell stress is shown in Figure S13A. It is proven that there is a large electrostatic force acted upon droplet front side, so that droplet would move forward. The electrostatic acted on droplet at different Y-position could be obtained. The ratio of electrostatic force F_e and maximum electrostatic force F_{emax} at different position is plotted as shown in Figure S13B. It is

shown that the electrostatic force first increases and then decreases to a negative value, finally it decreases to about 0. Besides, as the applied negative voltage increases, the maximum electrostatic force occurs at a further position. It means that as the applied negative voltage increases, the droplet could be subjected to a larger force in a longer distance. Therefore, droplet moves faster when applied negative voltage is larger. As shown in Figure S14, it is also noted that the location of the highest potential not the location of the largest electrostatic force generated by the droplets. At the highest potential location, the droplet and its nearby area on film share the same polarity of charges, which decreases the net force. In contrast, when the droplet is located at the boundary of opposite charge polarity, the droplet and its two sides area on film have different polarity of charges, the net force is larger.

The ratio of the electrostatic force F_e to the maximum electrostatic force F_{emax} could also be negative. Based on the same foundation, when the front area of a droplet on dielectric film stores more charges than the rear area of a droplet on dielectric film, and the charges carried by the droplet share the same polarity, the net force is opposite to the droplet moving direction.

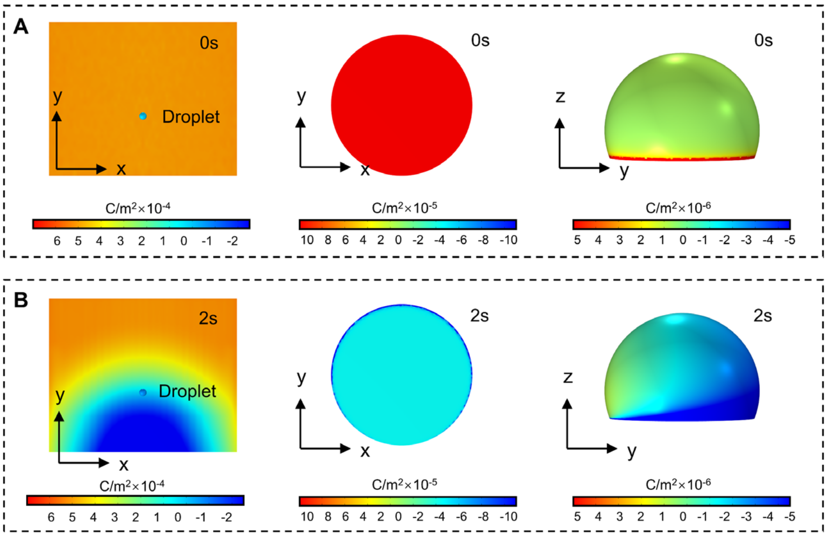

Figure S12. The simulation results of electrostatic force using COSMOL software. (A) The polymer and droplet surface charge distributions when there is only positive deposition. (B) The polymer and droplet surface charge distributions when there is a 2-s duration negative charges deposition after positive charges are deposited.

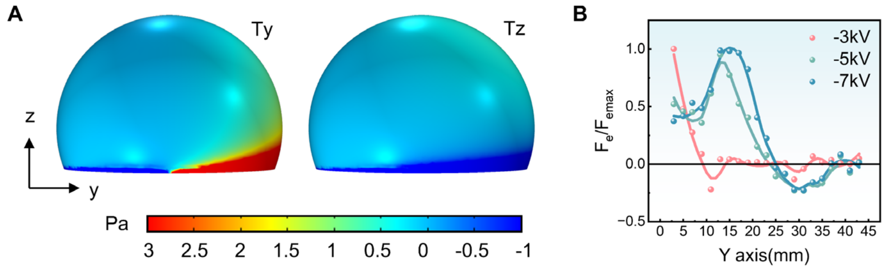

Figure S13. Electrostatic force calculation. (A) Maxwell stress of droplet surface. (B) F_e/F_{emax} varies as Y axis.

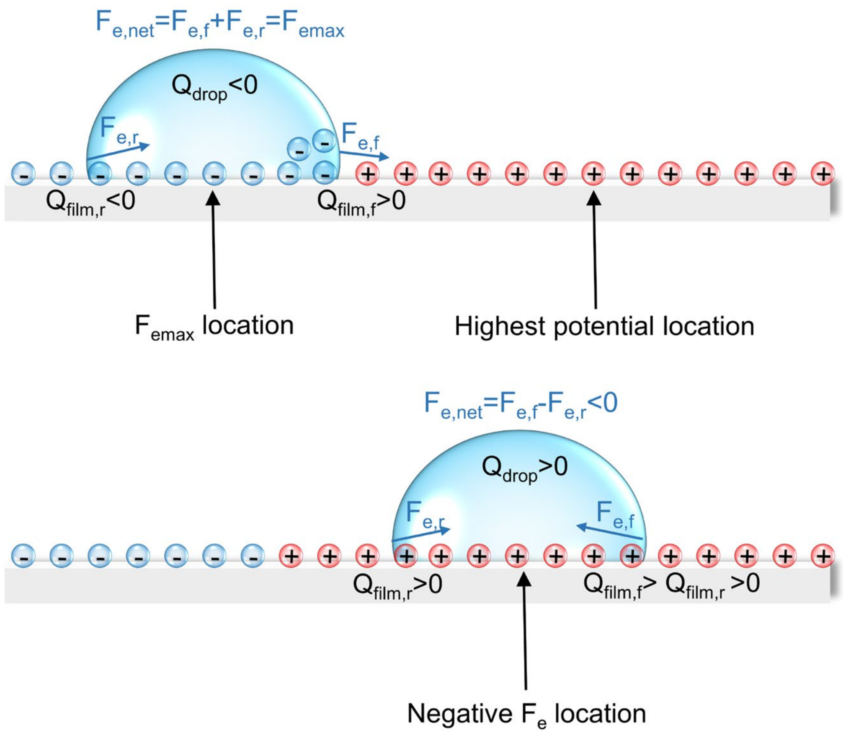

Figure S14. The schematic illustration of location of F_{emax}, Highest potential and Negative F_e. The Q_{drop}, $Q_{film,r}$, $Q_{film,f}$ represent charge carries by droplet, film area rear

of droplet and front of droplet. The $F_{e,r}$, $F_{e,f}$, $F_{e,net}$ represent electrostatic force acted on rear part of droplet and front part of droplet, and net electrostatic force.

3. Droplet acting force analysis

Various resistance force would prevent droplet from achieving a high-speed and universal motion. Here, our mechanism supplies a significant electrostatic force to overcome the following resistance force.

3.1 Lateral adhesion force

Lateral adhesion force prevents droplet from starting off on surfaces with large CAH. In order to propel droplet, the electrostatic force should be at least larger than the lateral adhesion force, which is related to CAH, and the adhesion force could be calculated by the following equation[4]:

$$F_{ad} = \frac{\pi}{2}\gamma l(cos\theta_r - cos\theta_a) \quad (7)$$

where F_{ad} is the lateral adhesion force, γ is the interfacial tension of the liquid and gas, l is the contact width. θ_a and θ_r are the advancing contact angle and receding contact angle, respectively.

The CAH is different in most cases in which different droplet is on different polymer surface. Therefore, the lateral adhesion force is different as well. The measured CAH and calculated adhesion force is listed in Table S2. It is shown that the F_{ad} overcome by our mechanism varies from 15 μN to 230 μN. The adhesion force reflects the quantity of electrostatic force to a great degree. The maximum electrostatic force acted on a 10-μL droplet is at least reaches up to 230 μN, the F_{ad} of droplet on acrylic surface.

3.2 Friction resistance force analysis

Droplet friction resistance force prevents droplet from achieving a high speed on surface without modification. When droplet continues moving on polymer surface, droplet is also constantly subjected to a non-negligible friction resistance force which

is usually related to droplet volumes and velocity. The friction force is measured using a micropipette-based method as shown in Figure S15A. A micropipette is put into a droplet which is placed at polymer surface. When the surface is moving at a preset velocity, the micropipette will have a deflection, then the friction force could be calculated based on the deflection by the following equation:

$$F = k\Delta x \tag{8}$$

where F is the friction, k is the coefficient, which could be obtained measuring the deflections caused by droplet gravity. Δx is the deflection.

Based on this method, the droplet friction resistant is measured and the result is shown in Figure S15B. It shows that the friction resistance force increases when droplet volume and velocity increases.

The result shows that when droplet moves as a velocity over 20 mm/s, the droplet is subjected to a large force (about 30 μN). Thanks to the large electrostatic force of dynamic surface-charge wetting, the droplet could achieve an average velocity of about 130 mm/s (Figure 1C).

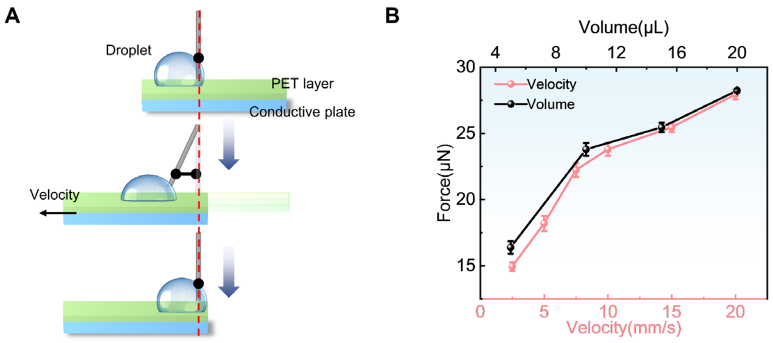

Figure S15. Friction resistance force measurement. (A)Schematic illustration of measurement set up. (B)Friction resistance force variation as droplet velocity and volume.

3.3 Barrier resistance force analysis

The various barriers generate a resistance force prevent droplet from achieving motion on heterogeneous surface, which makes droplet propulsion less adaptable. The droplet motion on surfaces with the edge and anti-wettability barriers was tested, the schematic illustration that droplet break the barriers is shown in Figures S16A-B. The time-dependent displacement is shown in Figures S16C-D. The droplet would be subjected to a large resistance when it meets the barriers, the force could be measured using micropipette method as well, as shown in Figures S15E-H. It is noted that the anti-wettability barrier resistance force could also be calculated using equation (7). The contact angles in two areas are regarded as θ_a and θ_r respectively.

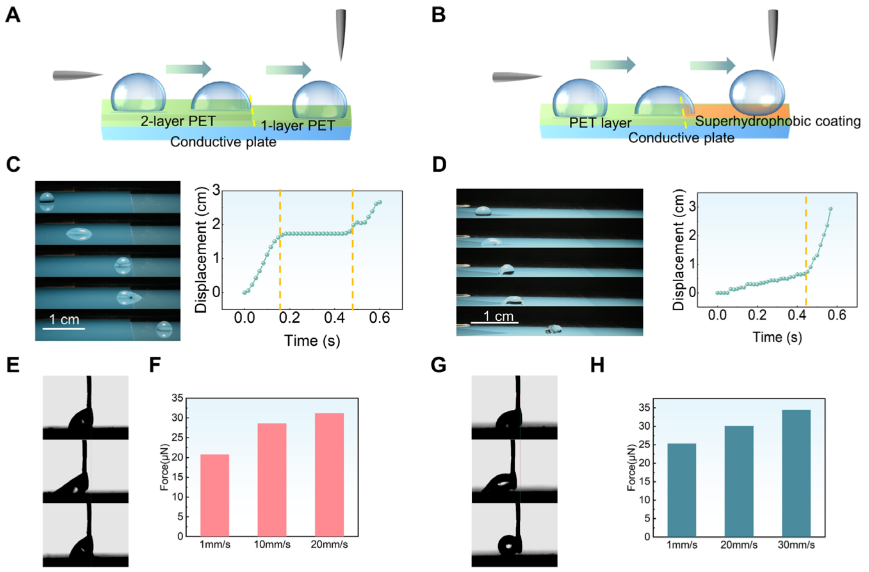

Figure S16. Barrier resistance force analysis. (A) The schematic illustration of droplet break edge barrier. (B) The schematic illustration of droplet breaking anti-wettability gradient barrier. (C) Sequential images of a 20-μL water droplet overcoming edge barrier (Left). The time-dependent displacement of droplet motion. (D) Sequential images of a 20-μL water droplet overcoming anti-wettability gradient barrier (Left). The time-dependent displacement of droplet motion. (E) The sequential images of measuring edge barrier resistance force. (F) The measured edge barrier

resistance force at different droplet velocity. (G) The sequential images of measuring anti-wettability gradient barrier resistance force. (H) The measured anti-wettability gradient barrier resistance force at different droplet velocity.

3.4 Ratio of electrostatic force and gravity calculation

Taken the above measurement and calculation together, the magnitude of electrostatic force could be achieved, we use the ratio R_F between F_e and gravity of droplet to exclude the impact of droplet volume. Based on the above discussion, the electrostatic force could be indirectly gained by the lateral adhesion force (F_{ad}) and barrier resistance force (F_b). For electrostatic force should be at least larger than the resistance force so that droplet could be propelled, the maximum F_{ad} and F_b was regarded as the value of the electrostatic force. The ratio F_{ad}/mg and F_b/mg are regarded as R_{F1} and R_{F2}, and the calculated value is listed in Table S2. It is proven that the driving force could reach about 3-6 times of gravity.

4. Mechanism analysis of high-performance droplet handling through spatially and temporally controlling the charge deposition

4.1 Droplet repeated motion mechanism

The temporally charge deposition controlling enables droplet move repeatedly. The mechanism is shown in Figure S17. Firstly, the right needle emits positive charges to the polymer surface and the surface is sufficiently positive charged, secondly, the left needle emits negative charges to surface and droplet moves forward. At this time the surface is sufficiently negative charged. Therefore, by repeatedly applying opposite charges on right needle, unlimited times deposition of oscillating and opposite surface charges is achieved to manipulate the droplet move back and forth all the time.

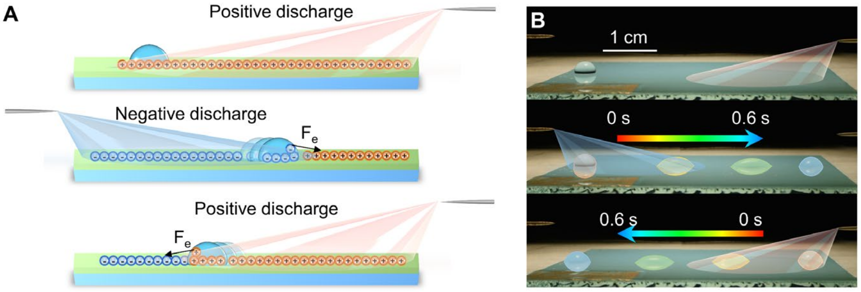

Figure S17. Droplet repeated propulsion. (A) The schematic illustration of droplet repeated propulsion mechanism. (B) The time-lapse trajectory of a 5-μL droplet repeated propulsion.

4.2 Droplet transport along preset track

The temporally charge deposition controlling enables droplet move along preset track. Firstly, a simple experiment is conducted to verity that droplet would move to the charged area (Figure S18). A grounded conductive plate pattern covers half of the polymer surface, under corona discharge, only the uncovered surface area could be charged. Under negative discharge, droplet is subjected to an electrostatic force

towards charged area, droplet therefore reaches the charged area. Based on this mechanism, the on-demand droplet motion track could be achieved.

We modify the plate electrode to a pattern with three channels and put it under polymer surface. After corona discharge, positive charges are deposited on the three channels. Then, by applying negative discharge, three droplets move along their channels and finally merge (Figure S19).

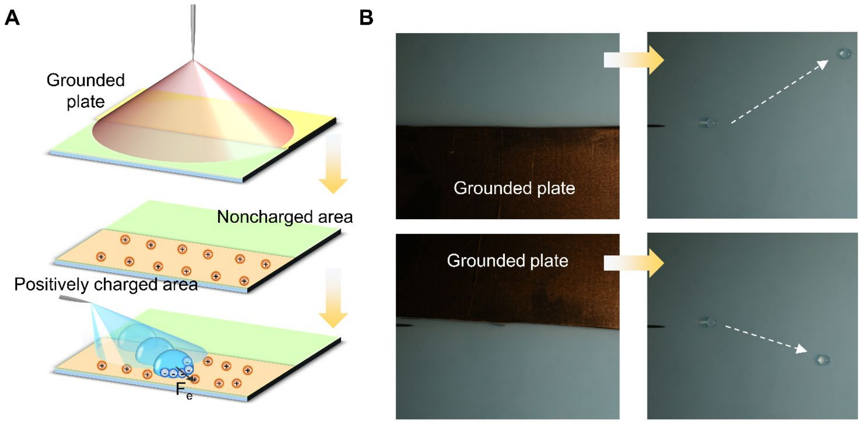

Figure S18. Droplet track transport on a half-charged surface. (A) The schematic illustration of regional charging. (B) The time-lapse trajectory of a 5-μL droplet moving under the impact of regional deposited charges.

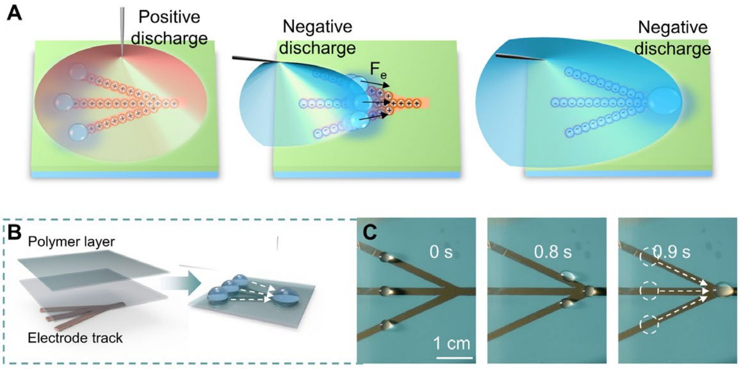

Figure S19. Droplet transport along certain track. (A) The schematic illustration of charged track. (B) The time-lapse trajectory of a 2-μL droplet moving along the preset track.

4.3 Droplet infinite distance transport mechanism

The spatiotemporally charge deposition controlling enables droplet infinite distance transport. As shown in Figure S20, three vertical needles are listed in line with a 10-mm interval. The middle needle first emits positive charges to the middle aera of polymer surface, then the first needle emits negative charges to the droplet and its near region. Negative charged droplet then moves to the area beneath the middle needle. Then this process is repeated, the third needle emits positive charges to the region below it, after that, the middle needle emits negative charges to the droplet and its near region, the droplet would move to the area beneath the third needle. For the needles with any number could be listed like this mode, the droplet could move with infinite distance.

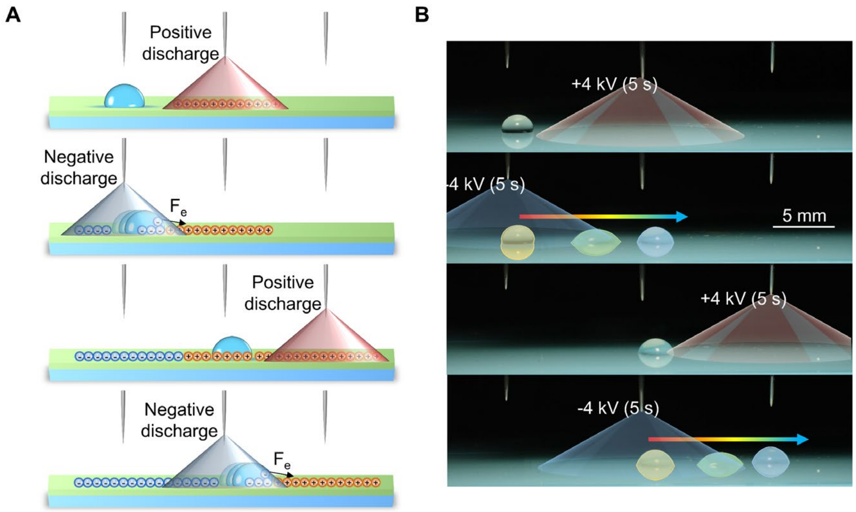

Figure S20. Droplet infinite distance transport (A) The schematic illustration of droplet infinite distance transport. (B) Sequential images of 7-μL droplet sustainable motion under sequentially applying voltages of three vertical needle electrodes.

4.4 Droplet swerve mechanism

The spatiotemporally charge deposition controlling also enables droplet a direction swerve function. This manipulation could be simply completed by three

orthogonal needles, as shown in Figure S21. Like droplet propulsion in a straight trajectory, positive charges are firstly deposited on the whole polymer surface by applying positive voltage on a vertical needle. Then, by applying negative voltage on a horizontal needle, the droplet could move forward along the direction parallel to the horizontal needle. Then, by applying negative voltage on another horizontal needle perpendicular to it, the droplet could move along another direction.

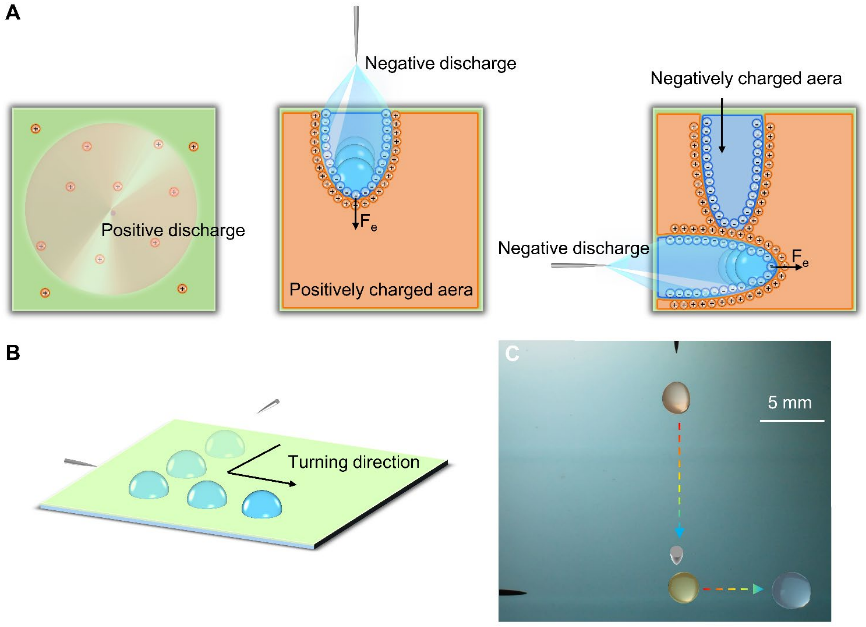

Figure S21. Droplet swerve manipulation. (A) The schematic illustration of droplet swerve mechanism. (B) The schematic illustration of droplet swerve. (C) The time-lapse trajectory of a 5-μL droplet moving and change its direction.

4.5 Programmable droplet microreaction

As shown in Figure S22, we sequentially propel droplet by depositing charges on different area on film in sequence. The charges form a track to propel certain droplet move forward. It is noted that the charges are only deposited where conductive are located under the film. Therefore, the controllable depositing area is achieved by sequentially rotating conductive bar and inducing corona discharge. The specific steps and mechanism are shown in schematic illustration.

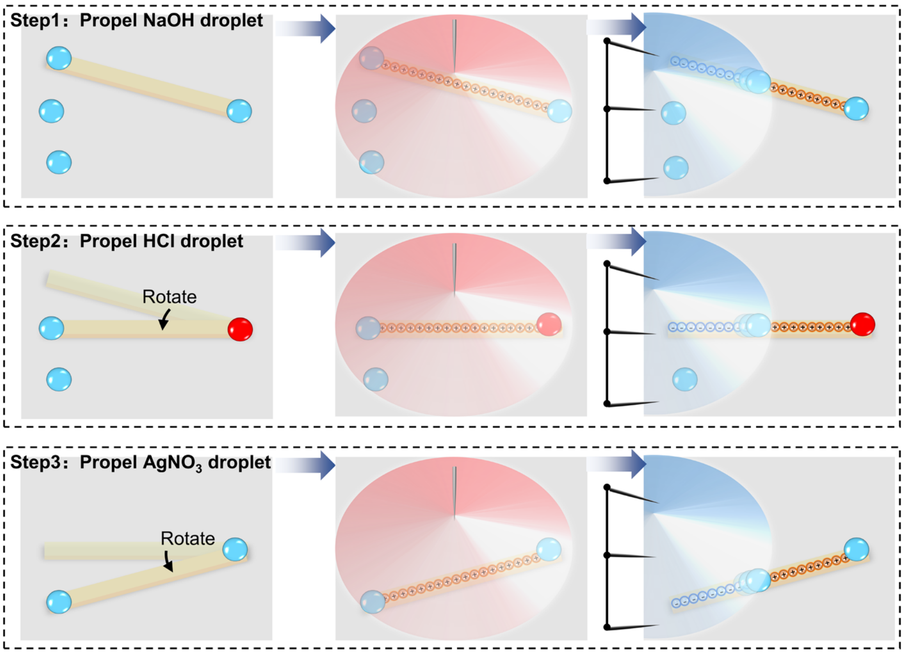

Figure 22. Schematic illustration of adjustable and continuous droplet microreactions.

Table S1. Equilibrium contact angles, advancing and receding contact angles.

	Equilibrium contact angle/°	Advancing contact angle/°	Receding contact angle/°	CAH/°	Lateral adhesion force (10-μL) /μN
Water on kapton	100.1	101.5	77.9	23.6	143.5
Water on PET	105.5	107.3	95.7	11.6	65.8
Water on BOPP	99.3	107.2	98.2	9	54.1
Water on acrylic	85.8	91.1	56	35.1	230.9
Water on PTFE	102.1	103.3	97.2	6.1	36.0
Ethanol on PTFE	39.7	47	30.9	16.1	103.3

Table S2. Values of different forces acted on 1-μL droplet.

	Lateral adhesion force/μN	Friction force/μN	Gravity/μN	Ratio of driving force to gravity
PET	30.5	20.2	10	3.1
Edge structure barrier		31	10	3.1
Wettability gradient barrier	62.3	34	10	6.2

Captions for Supplementary Movies

Movie S1. Droplet propulsion on diverse solid surfaces.

Movie S2. Droplet motion with high time resolution.

Movie S3. Droplet breaks transport barriers.

Movie S4. High-performance droplet handling.

Movie S5. Versatile droplet-propulsion applications.

References

1. Pierce, E., Carmona, F. J., Amirfazli, A. Understanding of sliding and contact angle results in tilted plate experiments. *Colloids Surf. A* **323,** 73-82 (2008).

2. Zhong, Y., Kou, R., Wang, M., Qiao, Y. Electrification mechanism of corona charged organic electrets *J. Phys. D-Appl. Phys.* **52,** 445303 (2019).

3. https://www.comsol.com/multiphysics/electrostatics-theory.

4. Gao, N. et al. How drops start sliding over solid surfaces. *Nat. Phys.* **14,** 191-196 (2018).
